# The oxygen sensor MgFnr controls magnetite biomineralization by regulation of denitrification in *Magnetospirillum gryphiswaldense*

**DOI:** 10.1186/1471-2180-14-153

**Published:** 2014-06-10

**Authors:** Yingjie Li, Monique Sabaty, Sarah Borg, Karen T Silva, David Pignol, Dirk Schüler

**Affiliations:** 1 Department Biologie I, Mikrobiologie, Ludwig-Maximilians-Universität München, Großhaderner Str. 2-4, 82152 Planegg-Martinsried, Germany; 2 CEA, IBEB, Laboratoire de Bioénergétique Cellulaire, Saint-Paul-lez-Durance F-13108, France; 3 CNRS, UMR Biologie Végétale & Microbiologie Environnementales, Saint-Paul-lez-Durance F-13108, France; 4 Aix-Marseille Université, Saint-Paul-lez-Durance F-13108, France; 5 Lehrstuhl f. Mikrobiologie, Universität Bayreuth, 95447 Bayreuth, Germany

**Keywords:** *Magnetospirillum gryphiswaldense*, Magnetite biomineralization, Oxygen regulation, Denitrification, Oxygen sensor Fnr

## Abstract

**Background:**

Magnetotactic bacteria are capable of synthesizing magnetosomes only under oxygen-limited conditions. However, the mechanism of the aerobic repression on magnetite biomineralization has remained unknown. In *Escherichia coli* and other bacteria, Fnr (fumarate and nitrate reduction regulator) proteins are known to be involved in controlling the switch between microaerobic and aerobic metabolism. Here, we report on an Fnr-like protein (MgFnr) and its role in growth metabolism and magnetite biomineralization in the alphaproteobacterium *Magnetospirillum gryphiswaldense*.

**Results:**

Deletion of *Mgfnr* not only resulted in decreased N_2_ production due to reduced N_2_O reductase activity, but also impaired magnetite biomineralization under microaerobic conditions in the presence of nitrate. Overexpression of MgFnr in the WT also caused the synthesis of smaller magnetite particles under anaerobic and microaerobic conditions in the presence of nitrate. These data suggest that proper expression of MgFnr is required for WT-like magnetosome synthesis, which is regulated by oxygen. Analyses of transcriptional *gusA* reporter fusions revealed that besides showing similar properties to Fnr proteins reported in other bacteria, MgFnr is involved in the repression of the expression of denitrification genes *nor* and *nosZ* under aerobic conditions, possibly owing to several unique amino acid residues specific to MTB-Fnr.

**Conclusions:**

We have identified and thoroughly characterized the first regulatory protein mediating denitrification growth and magnetite biomineralization in response to different oxygen conditions in a magnetotactic bacterium. Our findings reveal that the global oxygen regulator MgFnr is a genuine O_2_ sensor. It is involved in controlling expression of denitrification genes and thereby plays an indirect role in maintaining proper redox conditions required for magnetite biomineralization.

## Background

Magnetotactic bacteria (MTB) use magnetosomes for orientation in the Earth’s magnetic field to search for growth-favoring oxygen-limited zones of stratified aquatic habitats [1]. In the freshwater alphaproteobacterium *Magnetospirillum gryphiswaldense* (in the following referred to as MSR-1) and other MTB, magnetosomes are membrane-enveloped magnetic crystals of magnetite (Fe_3_O_4_) that are aligned in chains [1]. Magnetite biomineralization is not only controlled by more than 30 specific genes encoded within a genomic magnetosome island (MAI) [2-4], but also requires genes located outside MAI for synthesis of WT-like magnetosomes [5,6]. Although the mechanism of biomineralization is not completely understood, it has been proposed that the biosynthesis of mixed-valence iron oxide magnetite [FeII(FeIII)_2_O_4_] occurs by coprecipitation of ferrous and ferric iron in supersaturating concentrations, which requires a balanced ratio of ferrous and ferric iron [7-9]. In magnetospirilla, magnetosome formation is only induced at low oxygen tension, and maximum magnetosome yield was found under microaerobic conditions in the presence of nitrate, whereas aerobic conditions completely inhibit magnetite biomineralization [5,10]. However, it is unknown whether this aerobic repression is controlled by biological regulation, or alternatively, directly caused by chemical oxidation of iron ions within the cells. In addition, our recent work indicated that magnetite biomineralization in MSR-1 is linked to denitrification [5,6]. Deletion of *nap* genes encoding a periplasmic nitrate reductase not only abolished anaerobic growth and delayed aerobic growth in both nitrate and ammonium medium, but also severely impaired magnetite biomineralization and resulted in biosynthesis of fewer, smaller and irregular crystals during denitrification and microaerobic respiration [5]. In addition, loss of the nitrite reductase gene *nirS* led to defective growth of cells, which synthesized fewer, smaller and irregular crystals during nitrate reduction [6]. Transcriptional *gusA* fusions revealed that expression of *nap* is upregulated by oxygen, whereas other denitrification genes including *nirS*, *nor*, and *nosZ* display the highest expression under microaerobic conditions in the presence of nitrate [5].

In many bacteria, changes in oxygen tension serve as an important environmental signal to trigger adaptive changes between anaerobic and aerobic respiration. This has been well studied in *Escherichia coli* where oxygen deprivation induces the synthesis of a number of enzymes, particularly those carrying out anaerobic respiration [11-15]. The alteration of gene expression to facilitate the changes in energy metabolism is achieved by Fnr (fumarate and nitrate reduction regulator), a global anaerobic regulator under anaerobic or microaerobic conditions [16,17]. Fnr is a member of a superfamily of transcriptional sensors sharing sequence homology with the cyclic-AMP receptor class of proteins [18]. Like all members of this family, Fnr protein comprises a C-terminal DNA-binding domain involved in site-specific DNA recognition of target promoters, and an N-terminal sensory domain [12]. In *E. coli*, the sensor domain contains five cysteines, four of them (Cys-20, 23, 29, and 122) are essential and bind either a [4Fe-4S]^2+^ or a [2Fe-2S]^2+^ cluster [19-21]. Under anaerobic conditions, the Fnr protein is folded as a homodimer that contains one [4Fe-4S]^2+^ cluster per monomer. The Fnr dimers are able to bind target promoters and regulate transcription. Exposure of the [4Fe-4S]^2+^ clusters to oxygen results in its conversion to a [2Fe-2S]^2+^ oxidized form, which triggers conformational changes and further induces the protein monomerization and prevents its binding to DNA [22-28].

In the metabolically versatile MTB so far no oxygen regulators have been identified, and it is unknown how growth metabolism and magnetite biomineralization are regulated in response to different oxygen concentrations. Here, we for the first time identified a putative oxygen sensor MgFnr protein and analyzed its role in magnetite biomineralization. We showed that the MgFnr protein is involved in regulating expression of all denitrification genes in response to different oxygen concentrations, and thus plays an indirect role in magnetosome formation during denitrification. Although sharing similar characteristics with Fnr of other bacteria, MgFnr is able to repress the transcription of denitrification genes (*nor* and *nosZ)* under aerobic conditions, possibly owing to several unique amino acid residues specific to MTB-Fnr.

## Results

### Deletion of *Mgfnr* impairs biomineralization during microaerobic denitrification

**Figure 1:**
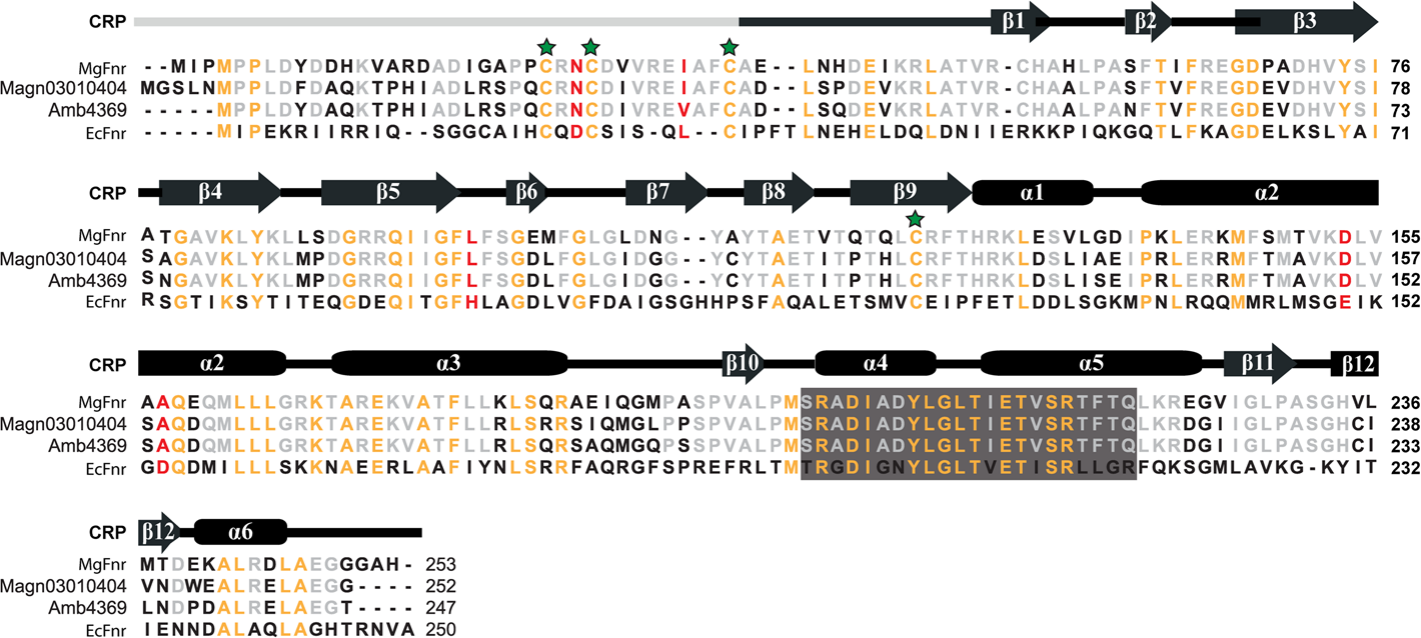
**Sequence alignment of Fnr proteins from different bacteria and proposed domain structure of one subunit of Fnr based on the structure of its homolog Crp from*E. coli*.** Conserved residues are shown in orange while residues which are only conserved in magnetospirilla are indicated in gray. In MSR-1, the first 37 amino acids that are absent in Crp contain three of the four Cys (Cys25, Cys28, Cys37, and C125 indicated by green stars) which ligate the [4Fe-4S] cluster. Gray boxes indicate DNA-binding motif. Single residue changes which are capable to activate transcription of nitrate reductase genes under aerobic conditions in *E. coli* are shown in red. Amb4369 is from *M. magneticum* strain and Magn03010404 is from *M. magnetotacticum*.

Using *E. coli* Fnr (hereafter referred to as EcFnr, GenBank accession no. AAC74416.1) as a query, we identified one putative Fnr protein, named MgFnr (Mgr_2553), encoded in the genome of MSR-1 (Figure 1). MgFnr has a higher similarity to Fnr proteins from other magnetospirilla, including Amb4369 from *Magnetospirillum magneticum* strain and Magn03010404 from *Magnetospirillum magnetotacticum* (76% identity, 97% similarity), than to EcFnr (28% identity, 37% similarity). Nevertheless, the MgFnr contains all signatory features of the Fnr family proteins: a C-terminal helix-turn-helix DNA binding domain and an N-terminal sensory domain containing the four cysteines (C25, C28, C37, and C125) found to be essential in EcFnr (Figure 1) [19].

**Figure 2:**
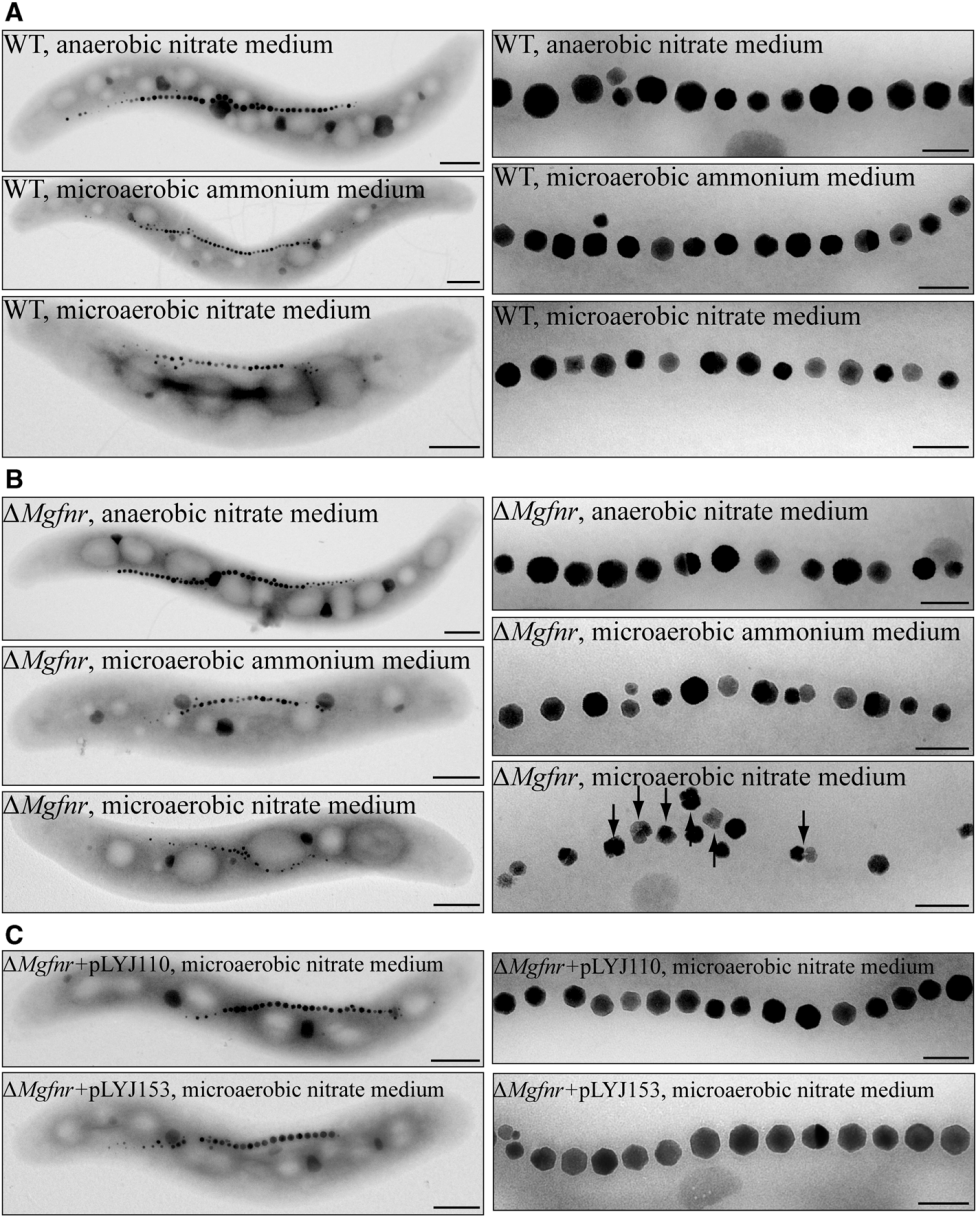
**Effects of*Mgfnr*deletions on magnetosome formation. (A)** Left: TEM images of whole cells of WT (from top to bottom) in anaerobic nitrate medium, microaerobic ammonium medium, and microaerobic nitrate medium. Bar, 500 nm. Right: Closeup views of magnetosome crystals shown on the left. Bar, 100 nm. **(B)** Left: TEM images of whole cells of Δ*Mgfnr* mutant (from top to bottom) in anaerobic nitrate medium, microaerobic ammonium medium, and microaerobic nitrate medium. Bar, 500 nm. Right: Closeup views of magnetosome crystals shown on the left. Irregular shaped particles are indicated by black arrows. Bar, 100 nm. **(C)** Left: TEM images of Δ*Mgfnr* mutant complemented with plasmids pLYJ110 harboring *Mgfnr* gene and pLYJ153 harboring *Ecfnr* gene in microaerobic nitrate medium. Bar, 500 nm. Right: Closeup views of magnetosome crystals shown on the left. Bar, 100 nm.

**Figure 3:**
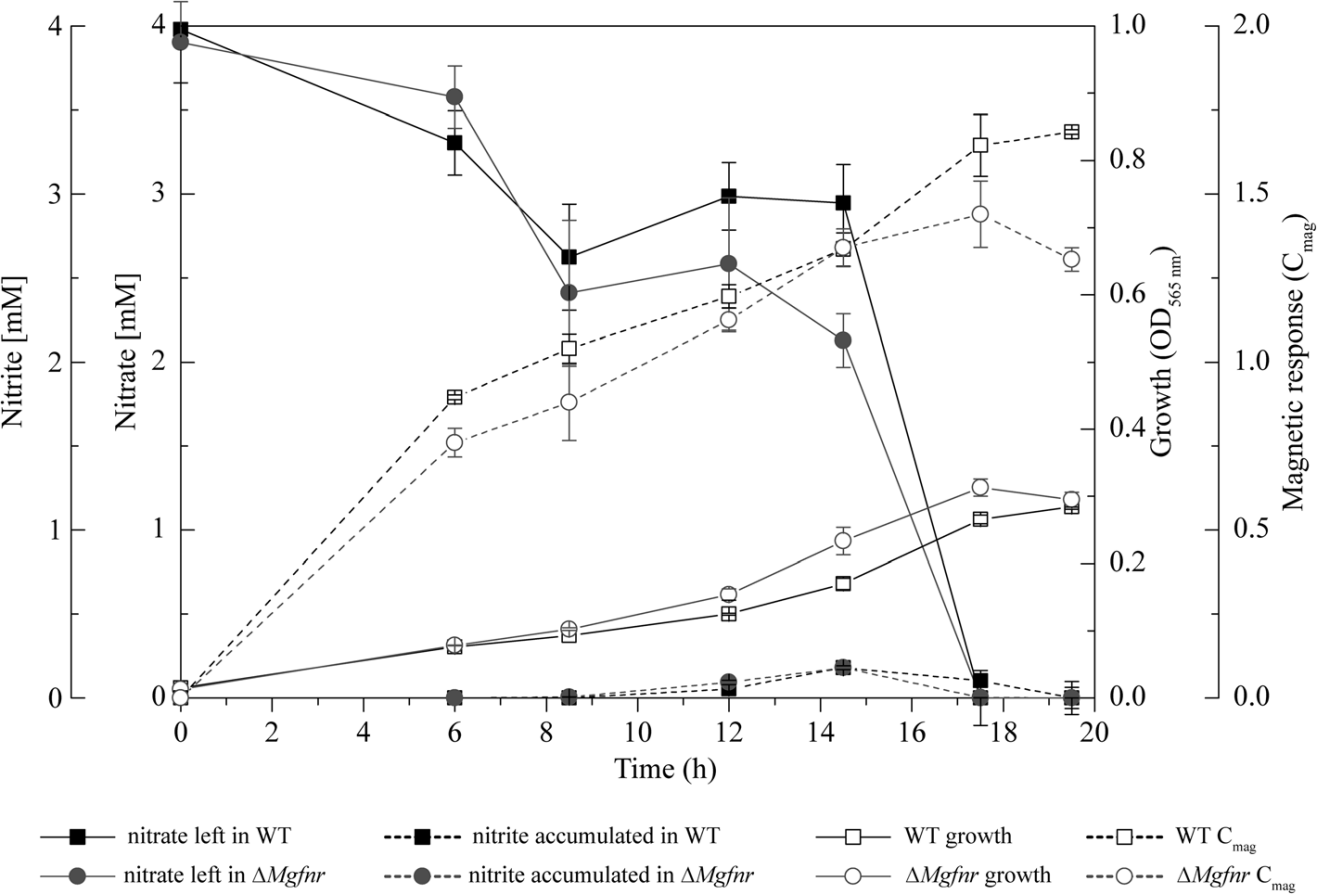
**Time courses of nitrate and nitrite utilization during microaerobic growth of WT and Δ*Mgfnr*mutant in nitrate medium.** Black square with solid line, nitrate left in WT; black square with dash line, nitrite accumulated in WT; gray circle with solid line, nitrate left in Δ*Mgfnr*; gray circle with dash line, nitrite accumulated in Δ*Mgfnr*; white square with solid line, growth of WT; white square with dash line, WT C_mag_; white circle with solid line, growth of Δ*Mgfnr*; white circle with dash line, Δ*Mgfnr* C_mag_.

We constructed an unmarked Δ*Mgfnr* mutant by a modified *cre-lox* based technique as described previously [29]. In both microaerobic ammonium medium and anaerobic nitrate medium, Δ*Mgfnr* mutant cells displayed WT-like growth and magnetic response (C_mag_) (data not shown) and produced WT-like magnetosome crystals (Figure 2A and B) with similar crystal size (40.2 ± 15.3 nm versus 38.0 ± 15.8 nm in WT under anaerobic conditions; 30.0 ± 13.6 nm versus 29.9 ± 14.5 nm in WT in microaerobic ammonium medium). However, although the Δ*Mgfnr* mutant grew as the WT in microaerobic nitrate medium, C_mag_ values were slightly lower than those in the WT during the entire growth (Figure 3). In agreement with this, Δ*Mgfnr* mutant cells contained smaller and aberrantly shaped particles in addition to particles with a WT-like size and appearance (Table 1, Figure 2B). Transcomplementation of Δ*Mgfnr* strain with the WT allele (Δ*Mgfnr* + pLYJ110) restored magnetosome formation back to the WT level with similar crystal size (Figure 2C, Table 1). However, WT overexpressing *Mgfnr* (WT + pLYJ110) produced smaller magnetite particles under anaerobic conditions (30.3 ± 15.1 nm, which was similar to that of WT in microaerobic nitrate medium) (Table 1, Additional file 1) and also under microaerobic conditions in the presence of nitrate (23.5 ± 13.8 nm versus 30.5 ± 12.4 in WT). This indicated that MgFnr is involved in magnetosome formation during nitrate reduction, and that the expression level of MgFnr is crucial for proper magnetite biomineralization.

**Table 1:** Crystal sizes in various strains under different conditions

**Strain**	**Anaerobic nitrate medium**	**Microaerobic nitrate medium**
WT	38.0 ± 15.8 nm	30.5 ± 12.4 nm
Δ*Mgfnr* mutant	40.2 ± 15.3 nm	21.9 ± 7.7 nm
WT + pLYJ110	30.3 ± 15.1 nm	23.5 ± 13.8 nm
Δ*Mgfnr* + pLYJ110	42.1 ± 21.9 nm	30.3 ± 22.3 nm
WT + pLYJ153	31.7 ± 18.7 nm	30.0 ± 21.6 nm
Δ*Mgfnr* + pLYJ153	40.9 ± 20.2 nm	31.3 ± 20.7 nm

### In Δ*Mgfnr* expression patterns of denitrification genes are different from those in WT

Deletion of *Mgfnr* resulted in impaired magnetite biomineralization only under microaerobic conditions in the presence of nitrate, suggesting a potential link to nitrate reduction. In addition, in *E. coli* and other bacteria, Fnr was shown to upregulate the expression of denitrification genes under microaerobic or anaerobic conditions [30,31]. Our earlier studies on MSR-1 showed that a complete denitrification pathway including genes encoding for nitrate (*nap*), nitrite (*nir*), nitric oxide (*nor*), and nitrous oxide reduction (*nos*) occurs for anaerobic growth. In addition, all denitrification genes in the WT were regulated by oxygen, and except for *nap*, which was upregulated by oxygen, the highest expression of other denitrification genes coincided with conditions permitting maximum magnetosome formation (e.g., low oxygen tensions and the presence of nitrate) [5]. Consistent with this, we found putative Fnr binding sites (*TTGAN*_6_*TCAA*) in the promoter regions of all operons involved in denitrification (Additional file 2). To gain insight whether these observed defects in magnetosome formation in Δ*Mgfnr* strain are indirectly caused by deregulation of denitrification genes, we analyzed the transcription of all denitrification genes by constructing *gusA* fusions in the Δ*Mgfnr* background (Table 2). In Δ*Mgfnr* strain, expression of *nap* was no longer upregulated by oxygen but displayed similar levels of β-glucuronidase activity under all tested conditions, which was higher than the maximum level in the WT. *nirS-gusA* showed a similar pattern as in WT, that is, it was upregulated by nitrate and downregulated by oxygen. However, an about 5-fold higher β-glucuronidase activity was measured under aerobic conditions compared to the WT. Δ*Mgfnr* mutant cells harboring the transcriptional *nor-gusA* reporter gene fusion exhibited a higher β-glucuronidase activity under microaerobic conditions in the presence of nitrate (416 U/mg) than in the absence of nitrate (151 U/mg), while it was lower than in the WT under the same conditions. However, oxygen did not inhibit the expression of *nor-gusA* in the Δ*Mgfnr* strain. Similarly, under microaerobic conditions, *nosZ-gusA* in the Δ*Mgfnr* strain also showed a higher β-glucuronidase activity in the presence of nitrate than in its absence, but compared to WT, expression of *nosZ-gusA* under microaerobic conditions in the presence of nitrate was decreased. Again, highest expression of *nosZ* was observed under aerobic conditions in the presence of nitrate. Taken together, these data indicated that deletion of *Mgfnr* resulted in a different oxygen-dependent regulation of denitrification genes, suggesting that MgFnr is involved in controlling the expression of denitrification and the observed defects in magnetosome formation in Δ*Mgfnr* mutant might indirectly result from loss of proper regulation of denitrification genes.

**Table 2:** **Effects of oxygen and nitrate on the expression of denitrification genes in** Δ ***Mgfnr*** **mutant**

**Promoter**	**Microaerobic conditions**		**Aerobic conditions**	
	**+ NO**_**3**_^**-**^	**- NO**_**3**_^**-**^	**+ NO**_**3**_^**-**^	**- NO**_**3**_^**-**^
*nap*	79.5 ± 41.8^a^	67.0 ± 29.4	79.6 ± 38.5	85.4 ± 30.9
	(16.2 ± 1.4)^b^	(15.9 ± 0.8)	(30.8 ± 2.6)	(28.6 ± 2.8)
*nirS*	266.3 ± 10.8	76.5 ± 28.3	85.4 ± 23.0	88.4 ± 54.9
	(124.0 ± 5.5)	(21.2 ± 9.6)	(14.2 ± 7.9)	(18.3 ± 7.8)
*nor*	414.7 ± 52.8	150.9 ± 52.4	559.7 ± 74.0	493.4 ± 52.9
	(762.8 ± 37.0)	(221.5 ± 52.4)	(204.4 ± 41.1)	(151.1 ± 10.5)
*nosZ*	327.8 ± 32.9	153.2 ± 62.5	751.3 ± 76.1	525.7 ± 53.6
	(519.0 ± 43.4)	(118.3 ± 33.3)	(146.6 ± 34.7)	(152.5 ± 21.9)

^a^Values of β-glucuronidase activity are averages and standard deviations for at least two replicate cultures. Units are recorded as nanomoles of product formed per minute per mg protein.

^b^Expression in the WT are shown in the “()” for comparison [5].

### Decreased N_2_ production in Δ*Mgfnr* mutant is due to lower N_2_O reductase activity

**Figure 4:**
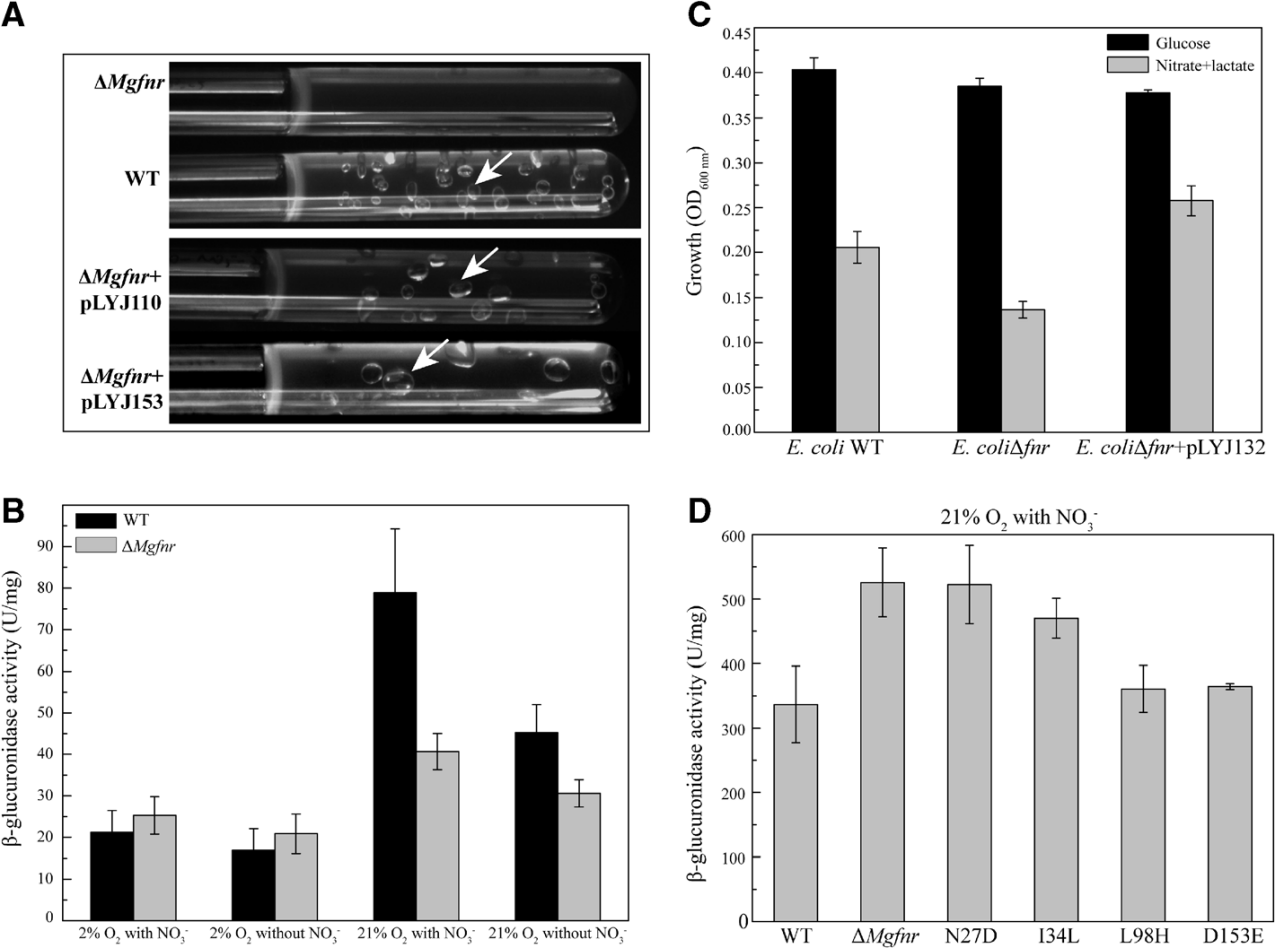
**Analysis of Δ*Mgfnr*mutant. (A)** N_2_ production in WT, Δ*Mgfnr* mutant, Δ*Mgfnr* mutant plus pLYJ110, and Δ*Mgfnr* mutant plus pLYJ153 cultures in oxygen gradient tubes with 0.3% agar. Δ*Mgfnr* mutant plus pLYJ110, and Δ*Mgfnr* mutant plus pLYJ153 cells contained respective *fnr* gene from MSR-1 and *E. coli*. Gas bubbles were indicated by white arrows. **(B)** Transcription of *Mgfnr* promoter fused to *gusA* in both WT and Δ*Mgfnr* mutant under different conditions. Expression was measured by β-glucuronidase activity. Cultures were grown aerobically or microaerobically in nitrate and ammonium medium. **(C)** Heterologous transcomplementation of Δ*Ecfnr* mutant harboring the plasmid pLYJ132 which contains *Mgfnr*. Cultures were anaerobically grown to stationary phase at 30°C in glucose minimal medium (black box) and lactate minimal medium (gray box). **(D)** Transcription of *nosZ* fused to *gusA* in *Mgfnr* variant strains under aerobic conditions in the presence of nitrate. Expression was measured by β-glucuronidase activity.

We next monitored the overall denitrification of MSR-1 WT and Δ*Mgfnr* mutant by growing cells in deep slush agar (0.3%) tubes containing nitrate medium in which entrapped gas bubbles are indicative for N_2_ production [5]. We found that although deletion of *Mgfnr* did not cause any growth defects under all tested conditions, in WT culture many N_2_ bubbles became visible after 24 h, while in Δ*Mgfnr* mutant only few bubbles were observed at any time of incubation, indicating that denitrification was reduced in this strain (Figure 4A). In contrast, the Δ*Mgfnr* complemented strain (Δ*Mgfnr* + pLYJ110) generated bubbles after 24 h as the WT. We therefore wanted to dissect at which step(s) of denitrification N_2_ production was affected. First, concentrations of nitrate and nitrite in microaerobic nitrate medium were measured during the entire growth of WT and Δ*Mgfnr* mutant to assess nitrate and nitrite reduction, which are catalyzed by Nap and NirS, respectively. As shown in Figure 3, no significant difference between WT and Δ*Mgfnr* mutant was observed for reduction of nitrate and nitrite. Nitrate disappeared slightly faster in the Δ*Mgfnr* mutant than in the WT, but this was not accompanied by an increased accumulation of nitrite. This meant that deletion of *Mgfnr* does not affect activities of the nitrate and nitrite reductase. We also measured the overall reduction of NO_3_^-^ to N_2_O and N_2_O to N_2_ by detecting the emission rate of respective reaction products in cell suspension at OD_565 nm_ of 1 with a gas-mass spectrometer (Table 3). For cultures grown in microaerobic ammonium medium, neither emission of N_2_O nor N_2_ was found in WT and the Δ*Mgfnr* mutant, suggesting that the presence of nitrate is essential to activate denitrification. After growth in microaerobic nitrate medium, N_2_O emission rates from nitrate were similar in WT and Δ*Mgfnr* mutant (Table 3). As estimated by N_2_ evolution, we found that N_2_O reductase activity was very low in both strains compared to nitrate, nitrite, and NO reductase activities, since the rate for N_2_O production from nitrate was 20-fold higher than the rate for N_2_ production. Due to the low values and the detection limit of the gas-mass spectrometer, the standard deviation is quite critical for evaluation of significance of the N_2_ emission values. However, in 8 independent experiments the N_2_ emission rates appeared lower for Δ*Mgfnr* strain than for the WT (0.4 μM/min versus 0.7 μM/min). In addition, we also tested oxygen reduction in both WT and Δ*Mgfnr* mutant grown under microaerobic conditions by determining the consumption rate of oxygen in cell suspension with the gas-mass spectrometer. WT and Δ*Mgfnr* mutant cells consumed oxygen at similar rates (Table 3), which indicated that MgFnr is not involved in regulation of O_2_ respiration.

**Table 3:** Rates of N_2_O and N_2_ emission in WT and Δ*Mgfnr* mutant after nitrate addition and rates of O_2_ consumption during aerobic respiration

**Culture (2% oxygen)**	**N**_**2**_**O emission**	**N**_**2**_**emission**	**O**_**2**_**consumption**
	**(μM/min**^**a**^**)**	**(μM/min)**	**(μM/min)**
WT without nitrate	ND^b^	ND	50.7 ± 10.0
Δ*Mgfnr* mutant without nitrate	ND	ND	44.0 ± 2.0
WT with nitrate	14.1 ± 2.0	0.7 ± 0.5	41.3 ± 2.0
Δ*Mgfnr* mutant with nitrate	12.0 ± 2.0	0.4 ± 0.2	44.0 ± 4.7

^a^The values (in μM per min for a cell suspension of OD_565 nm_ of 1) are the average of eight independent experiments. ^b^ND: not detectable.

### Expression of *Mgfnr* is increased by oxygen and upregulated by itself

Under aerobic conditions, the expression of denitrification genes *nor* and *nosZ* was upregulated in the Δ*Mgfnr* mutant, which suggested that MgFnr might be also active as repressor under aerobic conditions. Therefore, we first asked if transcription of the *Mgfnr* gene itself is under oxygen-dependent regulation. WT cells expressing *Mgfnr-gusA* showed the lowest β-glucuronidase activity under microaerobic conditions in the absence of nitrate, while the presence of nitrate slightly increased microaerobic expression of *Mgfnr* (Figure 4B). The expression of *Mgfnr* was induced approximately 4-fold in the presence of nitrate and more than 2-fold in the absence of nitrate under aerobic conditions relative to microaerobic conditions, which again suggested that MgFnr is likely active and acts as a repressor under aerobic conditions. In the Δ*Mgfnr* mutant, *Mgfnr-gusA* also exhibited the highest β-glucuronidase activity under aerobic conditions in the presence of nitrate. However, compared to WT under aerobic conditions, expression levels of *Mgfnr* in Δ*Mgfnr* mutant were significantly decreased, which indicated that expression of *Mgfnr* is also probably autoregulated. However, we failed to observe a putative Fnr binding site in the *Mgfnr* promoter region, implying other unknown proteins may be involved in the regulation of *Mgfnr*.

### MgFnr can complement *E. coli* Δ*Ecfnr* mutant

All previous observations were pointing towards a scenario, in which MgFnr may also repress expression of denitrification genes under aerobic conditions, which however has never been reported for any Fnr protein from other bacteria. Therefore, the question arose as to whether MgFnr is a genuine oxygen-responsive regulator. Consequently, an *Ecfnr* deletion mutant Δ*Ecfnr* was transcomplemented with *Mgfnr*. As shown before [11], Δ*Ecfnr* cells displayed deficient anaerobic growth when nitrate was used as the sole electron acceptor on lactate minimal medium, whereas they grew to similar yields as the WT anaerobically growing on glucose medium (Figure 4C). However, in the Δ*Ecfnr* + pLYJ132 strain which contained the WT-*Mgfnr* gene, anaerobic growth in the presence of nitrate was restored back to *E. coli* WT-like level, which demonstrated that MgFnr is also functional in *E. coli*. Vice versa, the MSR-1 Δ*Mgfnr* strain containing *Ecfnr* gene (Δ*Mgfnr* + pLYJ153) generated N_2_ bubbles after 24 h (Figure 4A), suggesting that EcFnr also functions in MSR-1. As shown in Figure 2C and Table 1, Δ*Mgfnr* + pLYJ153 strain containing *Ecfnr* again synthesized WT-like magnetite crystals. Under anaerobic conditions, overexpression of EcFnr resulted in a decrease in crystals size as overexpression of MgFnr does (Table 1, Additional file 1). However, when EcFnr was overexpressed in MSR-1 WT under microaerobic conditions, magnetite crystals with WT size were formed, contrary to what was observed with overexpression of MgFnr*.* Altogether, our observations suggested that MgFnr is a genuine oxygen sensor and displays an equivalent *in vivo* function as EcFnr in *E. coli*, but some functions of the MgFnr might be slightly distinct from the EcFnr.

### MgFnr mutations N27D and I34L increase expression of *nosZ* under aerobic conditions

In *E. coli*, it was observed that some single amino acid substitutions at positions not widely conserved among the Fnr family caused an increased stability of Fnr toward oxygen, and consequently, transcription of nitrate reductase genes became activated under aerobic conditions [25,30,32]. As shown in Figure 1, none of these reported amino acids in EcFnr (Asp-22, Leu-28, His-93, Glu-150, and Asp-154) is conserved in MgFnr (Asn-27, Ile-34, Leu-98, Asp-153, and Ala-157, respectively). However, the residues present in MgFnr are highly conserved among Fnr proteins from magnetospirilla except for MgFnr Ile-34 which is replaced by Val in *M. magneticum* Fnr. This indicates that some functional difference might occur between Fnr proteins from magnetospirilla and *E. coli*. Therefore, to test whether these sequence differences affect the stability of MgFnr to oxygen, we constructed several *Mgfnr* mutants, in which single amino acids of MgFnr were substituted by those present in EcFnr (N27D, I34L, L98H, and D153E) (Figure 1). With *nosZ* as an example, we measured β-glucuronidase activity of *nosZ-gusA* fusion in *Mgfnr* variant strains under different conditions. All MgFnr mutants exhibited decreased levels of *nosZ-gusA* (70%-90% of WT) expression in microaerobic nitrate medium (Additional file 3). Under aerobic conditions, N27D and I34L strains showed high *nosZ-gusA* expression, similar to that in Δ*Mgfnr* mutant, whereas L98H and D153E displayed the lowest expression which was similar to the WT (Figure 4D). We also investigated denitrification by N_2_ bubble formation of *Mgfnr* variant strains in deep slush agar tubes. Hardly any N_2_ was produced in all *Mgfnr* mutant strains (data not shown). All *Mgfnr* variant strains produced smaller magnetite particles and showed decreased iron concentrations and magnetic response (C_mag_ value) compared to the WT (Table 4, Additional file 4). However, the differences relative to the WT were more pronounced in the N27D and I34L strains, whose phenotypes were similar to those observed in Δ*Mgfnr* mutant (Table 4). This suggested that Asn-27 and Ile-34, which are located near Cys-28 and Cys-37, play an important role in maintaining a functional MgFnr.

**Table 4:** Measurements of C_mag_, iron content, and crystal size for various *Mgfnr* strains in microaerobic nitrate medium

**Strain**	**Magnetic response (C**_**mag**_**)**	**Iron content (%)**	**Crystal size (nm)**
WT	2.22 ± 0.01	100	29.3 ± 18.6
Δ*Mgfnr* mutant	1.78 ± 0.03	76.0 ± 0.06	20.7 ± 15.9
MgFnrN27D	1.77 ± 0.02	83.6 ± 0.03	19.2 ± 18.9
MgFnrI34L	1.83 ± 0.02	74.2 ± 0.07	21.3 ± 18.2
MgFnrL98H	1.91 ± 0.02	95.6 ± 0.16	24.3 ± 19.9
MgFnrD153E	1.93 ± 0.03	85.8 ± 0.14	23.6 ± 19.4

## Discussion

Our previous findings have implicated denitrification to be involved in redox control of anaerobic and microaerobic magnetite biomineralization [5,6]. In *E. coli* and other bacteria the switch between aerobic and microaerobic respiration such as nitrate reduction is primarily controlled by the Fnr regulator [16,17]. In this study, we have characterized the effect of the MgFnr protein on growth and magnetite biomineralization in MSR-1. Deletion of *Mgfnr* did not affect the growth yield, but impaired magnetosome formation under microaerobic conditions only in the presence of nitrate (i.e., when denitrification was active) but not in its absence. This implies that MgFnr might be involved in magnetite synthesis by regulation of denitrification genes, whereas expression of terminal oxidases for O_2_ respiration is likely not under the control of MgFnr, similar to Fnr from *Shewanella oneidensis*[33]. In fact, we found that neither the rates of oxygen consumption nor transcription of terminal oxidase genes [34] displayed any difference between the WT and Δ*Mgfnr* mutant. The presence of putative Fnr binding sites in the promoter regions of all operons of denitrification further indicates that MgFnr is involved in controlling the transcription of denitrification genes in response to different oxygen concentrations. Consistent with this, transcription patterns of denitrification genes in Δ*Mgfnr* mutant were different from WT. For example, in the Δ*Mgfnr* strain the expression of *nap* was no longer upregulated by oxygen, expression of *nirS* was much higher under aerobic conditions than WT, and aerobic expression of *nor* and *nosZ* was no longer repressed but upregulated by oxygen. Furthermore, we failed to identify a putative Fnr protein encoded in the genome of the nondenitrifying magnetotactic bacteria *Magnetococcus marinus* or *Desulfovibrio magneticus* strain RS-1, which also suggests that Fnr of MTB is likely only responsible to regulate genes encoding for denitrification, but not required for aerobic respiration. In addition, we also observed significantly decreased N_2_ evolution in deep slush agar tubes in Δ*Mgfnr* mutant. This raised the question at which step(s) of denitrification is affected by the loss of MgFnr. We propose that this is not likely caused by the reduction steps from NO_3_^-^ to N_2_O based on the following observations: (i) The consumption rate of NO_3_^-^ and NO_2_^-^ did not decrease in Δ*Mgfnr* mutant; (ii) NO is lethal to the cells while no defective growth was found in Δ*Mgfnr* mutant, and no NO emission was observed during mass spectrometry experiments which also implies that the activity of NO reductase is not decreased; (iii) The N_2_O emission rate after addition of nitrate was similar for Δ*Mgfnr* mutant and WT. Therefore, we conclude that loss of MgFnr affects the last step of denitrification, the reduction of N_2_O to N_2_. In agreement, the emission rate of N_2_ was lower for Δ*Mgfnr* mutant than for the WT. However, we cannot exclude the possibility that loss of MgFnr has an impact on further pathways involved in biomineralization other than denitrification. For instance, we have previously shown that (i) besides acting as nitrate reductase, Nap also plays a role in redox control for magnetosome formation, (ii) nitrite reductase NirS is capable to oxidize ferrous iron to ferric iron for magnetite synthesis, and (iii) NO reductase Nor also participates in magnetosome formation by yet unknown functions [5,6]. On the other hand, in the magnetotactic *Magnetovibrio blakemorei* strain MV-1 which is capable of anaerobic respiration with N_2_O as electron acceptor, a putative periplasmic Fe (II) oxidase was identified and proposed as N_2_O reductase NosZ [35], which suggests that N_2_O reductase might be also involved in magnetite biomineralization by unknown functions. In addition, in Δ*Mgfnr* mutant the different phenotypes observed under anaerobic and microaerobic conditions in the presence of nitrate indicate that MgFnr plays a more important role in magnetite biomineralization when O_2_ respiration and denitrification occur simultaneously. Our recent findings showed that maintaining a balance between aerobic respiration and denitrification is crucial for WT-like magnetite biomineralization [34]. In this case, MgFnr might provide the main contribution to mediate the expression of denitrification genes and therefore, poise the redox state for magnetosome formation.

Since deletion of *Mgfnr* altered oxygen-dependent regulation of denitrification genes under aerobic conditions, we hypothesized that MgFnr protein is active under aerobic conditions. Consistent with this, the expression of *Mgfnr* was upregulated by oxygen, which, however, was never reported for any Fnr protein from other bacteria. Studies on EcFnr mutants in *E. coli* have established the important role of a [4Fe-4S]^2+^ cluster in regulating EcFnr activity, and some single amino acid substitutions at positions not conserved in the Fnr family led to increased stability of Fnr to oxygen and activated transcription of nitrate reductase genes under aerobic growing conditions [24,25,30,32,36]. None of these reported amino acids of EcFnr are conserved in MgFnr, which might cause a more active MgFnr under aerobic conditions. Among them, Asn-27 and Ile-34 of MgFnr are located very closely to Cys-28 and Cys-37, two of the four cysteine residues that bind the [4Fe-4S]^2+^ cluster [37,38]. An *E. coli* EcFnr mutant protein containing amino acid substitution at either of these two positions showed increased expression of an EcFnr-dependent *lac* promoter under aerobic conditions [30,32,36]. In agreement with these observations, MgFnr mutants including N27D and I34L showed increased aerobic expression of *nosZ* promoter, suggesting that Asn-27 and Ile-34 of MgFnr are required for a functional MgFnr and likely play a role in maintaining the stability of [4Fe-4S]^2+^ cluster. However, MgFnr was able to complement Δ*Ecfnr* mutant back to WT-like growth, which indicates that MgFnr also has the universal properties of EcFnr. Nonetheless, Δ*Ecfnr* mutant and Δ*Mgfnr* mutant displayed significantly different phenotypes during anaerobic growth, such as a largely decreased growth yield in Δ*Ecfnr* mutant, but no defective growth in Δ*Mgfnr* mutant. These differences might be explained by different media used for cultivation because in *E. coli* deletion of *Ecfnr* only resulted in growth defect in some minimal media [11] while there is no minimal medium available, which provides reliable growth for MSR-1. In addition, not only deletion of *Mgfnr* but also overexpression of *Mgfnr* in WT affected anaerobic and microaerobic magnetite biomineralization in the presence of nitrate and caused the synthesis of smaller magnetosome particles, which indicates that the balanced expression of MgFnr is crucial for WT-like magnetosome synthesis and the expression level is under precise control, be regulated by oxygen. Therefore, MgFnr might play an important role in maintaining redox balance for magnetite synthesis by controlling the expression of denitrification genes, and thus the expression of MgFnr is required to be strictly regulated. On the other hand, since MgFnr serves as an activator for expression of denitrification genes (*nor* and *nosZ*) under microaerobic conditions while as a repressor on the same genes under aerobic conditions, it is proposed that other oxygen sensors involved in expression of *nor* and *nosZ* are regulated by MgFnr. For example, a NosR protein has been shown to be required to activate the transcription of *nos* gene in *Pseudomonas stutzeri*[39]. However, our data cannot rule out the possibility that MgFnr is also regulated by other yet unknown proteins and that other genes involved in magnetosome formation is controlled by MgFnr.

## Conclusions

We demonstrated for the first time that MgFnr is a genuine oxygen regulator in a magnetotactic bacterium and mediates anaerobic respiration. The expression of MgFnr is required to be precisely controlled, which is regulated by oxygen. In addition, MgFnr is also involved in regulation of magnetite biomineralization during denitrification, likely by controlling proper expression of denitrification genes. This allows the transcription to be adapted to changes in oxygen availability, and thus maintaining proper redox conditions for magnetite synthesis. Despite of general similarities with Fnr proteins from other bacteria, MgFnr is more insensitive to O_2_ and further displays additional functions for aerobic conditions, which might result from some non-conserved amino acids.

Although oxygen is known to be a major factor affecting magnetite biomineralization for decades, the mechanism of this effect in MTB is still unknown. The common observation that magnetosomes are only synthesized under oxygen-limited conditions raised the possibility of protein-mediated regulation of the biomineralization process. However, although MgFnr mediates oxygen-dependent regulation, its relatively subtle and indirect effects on magnetite biomineralization cannot account for the observed complete inhibition of magnetite biosynthesis under aerobic conditions. In addition to a possible effect caused by directly perturbing the redox balance of iron ions required for magnetite synthesis, another level of genetic regulation may exist in MSR-1. Since MgFnr only affects expression of denitrification genes but not genes encoding O_2_ respiration enzymes, magnetite biomineralization is also probably regulated by other unknown O_2_ sensors. Therefore, further research on respiratory pathways in MTB is likely to gain more insights into the mechanism of oxygen-dependent regulation of biomineralization.

## Methods

### Bacterial strains and growth conditions

Bacteria strains and plasmids used in this study are shown in Additional file 5. If not specified otherwise, *E. coli* strains were grown in lysogeny broth (LB) at 37°C, and MSR-1 strains were cultivated at 30°C in nitrate medium as described before [5]. In ammonium medium, nitrate was substituted by 4 mM ammonium chloride. When necessary, antibiotics were used at the following concentrations: *E. coli*: tetracycline (Tc), 12 μg/ml, kanamycin (Km), 25 μg/ml, and gentamicin (Gm), 15 μg/ml; MSR-1: Tc, 5 μg/ml, Km, 5 μg/ml, and Gm, 30 μg/ml. When *E. coli* strain BW29427 was used as donor in conjugation, 300 μM diaminopimelic acid (DAP) was added.

Experiments for growth and magnetic response (C_mag_) were monitored under microaerobic and anaerobic conditions in 250 ml flasks containing 100 ml media. For microaerobic conditions, flasks were sealed with butyl-rubber stoppers under a microaerobic gas mixture containing 2% O_2_ and 98% N_2_ before autoclaving. Anaerobic conditions were achieved by removing oxygen from gas mixture. For aerobic conditions, strains were cultured in free gas exchange with air in 300 ml flasks containing 20 ml medium agitated at 200 rpm. Optical density (OD) and magnetic response (C_mag_) were measured photometrically at 565 nm as previously described [40]. For gas production assay, cells were inoculated and mixed with nitrate medium with 0.3% agar in oxygen gradient tubes and exposed to the air.

### Genetic and molecular biology techniques

Standard molecular and genetic techniques were carried out for DNA isolation, digestion, ligation, and transformation [41]. All DNA products were sequenced using BigDye Terminator version 3.1 chemistry on an ABI 3700 capillary sequencer (Applied Biosystems, Darmstadt, Germany), and sequence data were analyzed with the software Vector NTI Advance® 11.5.1 (Invitrogen, Darmstadt, Germany). All oligonucleotide sequences used in this work are available if required.

### Construction of a MSR-1 Δ*Mgfnr* deletion mutant

All PCRs were performed using Phusion polymerase (NEB). Enzymes, including restriction enzymes and T4 DNA ligase, were purchased from Fermentas. To generate the unmarked Δ*Mgfnr* deletion mutant, a modified *cre-lox* method was used as previously described [29]. An about 2-kb downstream PCR fragment of *Mgfnr* was generated and cloned into NotI/EcoRI-digested pAL01 to obtain pLYJ106. The plasmid pLYJ106 was conjugationally integrated into the chromosome of MSR-1 and colonies screened positively by PCR for the presence of the kanamycin marker were designated Δ*Mgfnr-down* strain. Subsequently, the plasmid pLYJ105 containing a 2-kb upstream fragment of *Mgfnr* was integrated into the chromosome of Δ*Mgfnr-down* strain by conjugation. After verified by screening PCR for the presence of kanamycin and gentamicin markers, the strain was designated Δ*Mgfnr-up-down* strain. The *lox*-mediated excision of *Mgfnr* was initiated by conjugational transformation of pLYJ87 [6]. Precise excision was further confirmed by PCR amplification and sequencing. The plasmid pLYJ87 was lost by successive cultures in fresh nitrate medium. Finally, this strain was designated Δ*Mgfnr* mutant.

For genetic complementation of Δ*Mgfnr* mutant, the *Mgfnr* gene with its own promoter region was ligated into Acc65I/SacII-digested pBBR1MCS-2, yielding pLYJ110. Subsequently, pLYJ110 was transformed into MSR-1 WT and Δ*Mgfnr* mutant by conjugation. The *Ecfnr* gene from *E. coli K-12* was also hetero-complemented into Δ*Mgfnr* mutant and WT. The PCR fragment of *Ecfnr* from *E. coli* was digested with HindIII and XbaI and ligated into pLYJ36 to yield pLYJ153.

### Heterologous transcomplementation of an *E. coli* Δ*Ecfnr* mutant

First, Δ*Ecfnr* mutant with kanamycin marker was excised with the *E. coli* Quick and Easy gene deletion kit (Gene Bridges) and the Bac modification kit (Gene Bridges), as reported in [42]. This unmarked mutant was designated Δ*Ecfnr* mutant. To express MgFnr protein from MSR-1, *Mgfnr* was ligated into SmaI/XbaI-digested pBBR1MCS-2 to yield pLYJ132. Plasmid pLYJ132 was then transformed into Δ*Ecfnr* mutant. For transcomplementation analysis, strains were anaerobically grown in glucose minimal medium and lactate minimal medium [30].

### Construction of different *Mgfnr* variants

Substitutions at amino acid positions 27, 34, 98, and 153 were created by site-directed mutagenesis. First, PstI-SpeI digested fragment for each of substitutions was cloned into pOR093 to create pLYJ141 (*Mgfnr*-N27D), pLYJ142 (*Mgfnr*-I34L), pLYJ143 (*Mgfnr*-D153E), and pLYJ144 (*Mgfnr*-L98H), respectively. The different MgFnr mutants were subsequently obtained by a two-step homologous recombination technique in the same manner as described previously [43]. The *Mgfnr* variants were confirmed by PCR and sequencing.

### Analysis of transcriptional *gusA* fusions

To obtain the transcriptional *Mgfnr-gusA* fusion plasmid, *Mgfnr* promoter region was cloned into Acc65I/HindIII-digested pLYJ97, designated pLYJ109. To investigate the expression of *Mgfnr* under different conditions, β-glucuronidase activity was determined at 37°C as described before [5]. Units were recorded as nanomoles of product formed per minute per mg protein. Triplicate assays were measured and the values reported were averaged by using at least two independent experiments.

### Ferrozine assay

To determine the concentration of intracellular iron, cell pellet was washed twice with 1200 μl HEPES buffer (20 mM HEPES, 5 mM EDTA) to remove absorbed iron. After resuspended with 100 μl 65% HNO_3_, this mixture was incubated at 98°C for 3 h. When the sample cooled down to room temperature, 900 μl H_2_O was added for ferrozine assay as described before [44]. Briefly, the total Fe-content was determined by complete reduction of iron with hydroxylamine hydrochloride. This dissolved ferrous iron was further reacted with three ferrozine molecules to form an intensively purple-colour complex, which can be quantified spectrophotometrically at 562 nm.

### Nitrate and nitrite concentration assay

WT and Δ*Mgfnr* strains were grown under microaerobic conditions in the presence of nitrate. 1 ml culture at different time points was taken to detect nitrate and nitrite concentration as described in [5]. Nitrate was measured using Szechrome reagents (Polysciences, Inc.). Diluted 20-fold samples were mixed with equal modified Szechrome reagents and the absorbance recorded at 570 nm after 30 min. When nitrate was not detectable, cultures without dilution were detected to confirm the absence of nitrate. A nitrate standard curve (0–350 μM) was generated to convert absorbance values to concentrations. Nitrite was examined by the modified Griess reagent (Sigma). 100 μl diluted 20-fold samples of cultures were prepared and equal modified Griess reagent was subsequently added. The absorbance recorded at 540 nm after 15 min. When no nitrite was detected, cultures without dilution were detected to confirm the absence of nitrite. A nitrite standard curve (0–70 μM) was obtained to calculate final nitrite concentration. Duplicate assays were carried out and the values reported were measured in one representative experiment.

### Mass spectrometry measurements of O_2_ respiration and nitrate reduction

WT and Δ*Mgfnr* strains were grown under microaerobic conditions in the presence or absence of nitrate. The cells were centrifuged and resuspended in fresh ammonium medium. Then the suspension was placed in the measuring chamber (1.5 ml) of a mass spectrometer (model PrimaδB; Thermo Electron). The bottom of the chamber (Hansatech electrode type) was sealed by a Teflon membrane, allowing dissolved gases to be directly introduced through a vacuum line into the ion source of the mass spectrometer. The chamber was thermostated at 28°C, and the cell suspension was stirred continuously by a magnetic stirrer. For O_2_ respiration measurement, air was sparged into the suspension before chamber closing. The consumption of oxygen by the cells was followed at m/e = 32. For denitrification, the cells were sparged with Argon and nitrate reduction was measured using 2 mM K^15^NO_3_ (CEA 97.4% ^15^ N). NO, N_2_O and N_2_ concentrations were followed as a function of time.

### TEM and crystal analysis

If not specified, MSR-1 WT and mutants were grown at 30°C under anaerobic or microaerobic conditions for 20 h, concentrated and adsorbed onto carbon-coated copper grids. Samples were viewed and recorded with a Morgagni 268 microscope (FEI, Eindhoven, Netherlands) at 80 kV. For magnetosome analysis, more than 300 crystals were characterized for each strain.

### Sequence analysis

*fnr* genes were identified by BLASTP (http://www.blast.ncbi.nlm.nih.gov/Blast.cgi) homology searching in the genomes of MSR-1 (GenBank: CU459003.1), *M. magneticum* (GenBank AP007255.1), *M. magnetotacticum* (NCBI reference sequence NZ_AAAP00000000.1), *Mc. marinus* (GenBank accession number CP000471.1), and *D. magneticus* strain RS-1 (GenBank accession number AP010904.1). ClustalW was used for sequence alignment. The identification of Fnr binding sites in the promoter regions of the different operons encoding denitrification enzymes were performed with the virtual footprint software (PRODORIC, http://www.prodoric.tu-bs.de/vfp/index2.php).

## Competing interests

The authors declare that they have no competing interests.

## Authors’ contributions

YL and DS conceived and designed the research. YL, MS, SB, KS, and DP performed the experiments and analyzed the data. YL and DS wrote the manuscript. All authors read and approved the final manuscript.

## Supplementary Material

Additional file 1 **Magnetosome formation in WT overexpressing MgFnr.** Plasmid pLYJ110 and pLYJ153 contains *fnr* gene from MSR-1 and *E. coli*, respectively. Cells were grown in anaerobic nitrate medium. Bar, 100 nm. Click here for file

Additional file 2 **Detection of Fnr binding sites in the upstream regions of** ***nap*, *nirS*, *nor*, and *nosZ*.** The putative Fnr binding sites in the promoter regions are indicated in yellow. Click here for file

Additional file 3 **Transcription of** ***nosZ*** **fused to** ***gusA*** **in** ***Mgfnr*** **variant strains under microaerobic in the presence of nitrate.** Expression was measured by β-glucuronidase activity. Click here for file

Additional file 4 **Magnetosome formation in different** ***Mgfnr*** **variant strains.** Cells were grown in microaerobic nitrate medium. Bar, 100 nm. Irregular shaped particles are indicated by black arrows. Click here for file

Additional file 5 Bacterial strains and plasmids used in this work. Click here for file

## References

[B1] Jogler C Schüler D Genomics, genetics, and cell biology of magnetosome formation Annu Rev Microbiol 2009 63 501 521 10.1146/annurev.micro.62.081307.162908 19575557

[B2] Ullrich S Kube M Schübbe S Reinhardt R Schüler D A hypervariable 130-kilobase genomic region of *Magnetospirillum gryphiswaldense* comprises a magnetosome island which undergoes frequent rearrangements during stationary growth J Bacteriol 2005 187 7176 7184 10.1128/JB.187.21.7176-7184.2005 16237001 PMC1272989

[B3] Murat D Quinlan A Vali H Komeili A Comprehensive genetic dissection of the magnetosome gene island reveals the step-wise assembly of a prokaryotic organelle Proc Natl Acad Sci U S A 2010 107 5593 5598 10.1073/pnas.0914439107 20212111 PMC2851823

[B4] Lohsse A Ullrich S Katzmann E Borg S Wanner G Richter M Voigt B Schweder T Schüler D Functional analysis of the magnetosome island in *Magnetospirillum gryphiswaldense*: the *mamAB* operon is sufficient for magnetite biomineralization PLoS One 2011 6 e25561 10.1371/journal.pone.0025561 22043287 PMC3197154

[B5] Li YJ Katzmann E Borg S Schüler D The periplasmic nitrate reductase Nap is required for anaerobic growth and involved in redox control of magnetite biomineralization in *Magnetospirillum gryphiswaldense* J Bacteriol 2012 194 4847 4856 10.1128/JB.00903-12 22730130 PMC3430331

[B6] Li YJ Bali S Borg S Katzmann E Ferguson SJ Schüler D Cytochrome *cd*_1_ nitrite reductase NirS is involved in anaerobic magnetite biomineralization in *Magnetospirillum gryphiswaldense* and requires NirN for proper *d*_1_ heme assembly J Bacteriol 2013 195 4297 4309 10.1128/JB.00686-13 23893106 PMC3754751

[B7] Mann S Sparks NHC Board RG Magnetotactic bacteria: microbiology, biomineralization, palaeomagnetism and biotechnology Adv Microb Physiol 1990 31 125 181 2124779 10.1016/s0065-2911(08)60121-6

[B8] Faivre D Agrinier P Menguy N Zuddas P Pachana K Gloter A Laval J Guyot F Mineralogical and isotopic properties of inorganic nanocrystalline magnetites Geochim Cosmochim Acta 2004 68 4395 4403 10.1016/j.gca.2004.03.016

[B9] Faivre D Böttger LH Matzanke BF Schüler D Intracellular magnetite biomineralization in bacteria proceeds by a distinct pathway involving membrane-bound ferritin and an iron (II) species Angew Chem Int Ed Engl 2007 46 8495 8499 10.1002/anie.200700927 17902080

[B10] Heyen U Schüler D Growth and magnetosome formation by microaerophilic *Magnetospirillum* strains in an oxygen-controlled fermentor Appl Microbiol Biotechnol 2003 61 536 544 10.1007/s00253-002-1219-x 12764570

[B11] Lambden PR Guest JR Mutants of *Escherichia coli* K12 unable to use fumarate as an anaerobic electron acceptor J Gen Microbiol 1976 97 145 160 10.1099/00221287-97-2-145 796407

[B12] Spiro S Guest JR FNR and its role in oxygen-regulated gene expression in *Escherichia coli* FEMS Microbiol Rev 1990 6 399 428 2248796 10.1111/j.1574-6968.1990.tb04109.x

[B13] Tolla DA Savageau MA Phenotypic repertoire of the FNR regulatory network in *Escherichia coli* Mol Microbiol 2011 79 149 165 10.1111/j.1365-2958.2010.07437.x 21166900 PMC3075585

[B14] Tseng CP Albrecht J Gunsalus RP Effect of microaerophilic cell growth conditions on expression of the aerobic (*cyoABCDE* and *cydAB*) and anaerobic (*narGHJI*, *frdABCD*, and *dmsABC*) respiratory pathway genes in *Escherichia coli* J Bacteriol 1996 178 1094 1098 8576043 10.1128/jb.178.4.1094-1098.1996 PMC177770

[B15] Stewart V Bledsoe PJ Chen LL Cai A Catabolite repression control of *napF* (periplasmic nitrate reductase) operon expression in *Escherichia coli* K-12 J Bacteriol 2009 191 996 1005 10.1128/JB.00873-08 19060147 PMC2632075

[B16] Unden G Becker S Bongaerts J Holighaus G Schirawski J Six S O_2_-sensing and O_2_-dependent gene regulation in facultatively anaerobic bacteria Arch Microbiol 1995 164 81 90 8588737

[B17] Bueno E Mesa S Bedmar EJ Richardson DJ Delgado MJ Bacterial adaptation of respiration from oxic to microoxic and anoxic conditions: redox control Antioxid Redox Signal 2012 16 819 852 10.1089/ars.2011.4051 22098259 PMC3283443

[B18] Shaw DJ Rice DW Guest JR Homology between Cap and Fnr, a regulator of anaerobic respiration in *Escherichia coli* J Mol Biol 1983 166 241 247 10.1016/S0022-2836(83)80011-4 6343617

[B19] Green J Sharrocks AD Green B Geisow M Guest JR Properties of FNR proteins substituted at each of the five cysteine residues Mol Microbiol 1993 8 61 68 10.1111/j.1365-2958.1993.tb01203.x 8497198

[B20] Khoroshilova N Popescu C Munck E Beinert H Kiley PJ Iron-sulfur cluster disassembly in the FNR protein of *Escherichia coli* by O_2_: [4Fe-4S] to [2Fe-2S] conversion with loss of biological activity Proc Natl Acad Sci U S A 1997 94 6087 6092 10.1073/pnas.94.12.6087 9177174 PMC21006

[B21] Kiley PJ Beinert H Oxygen sensing by the global regulator, FNR: the role of the iron-sulfur cluster FEMS Microbiol Rev 1998 22 341 352 10.1111/j.1574-6976.1998.tb00375.x 9990723

[B22] Lazazzera BA Beinert H Khoroshilova N Kennedy MC Kiley PJ DNA binding and dimerization of the Fe-S-containing FNR protein from *Escherichia coli* are regulated by oxygen J Biol Chem 1996 271 2762 2768 10.1074/jbc.271.5.2762 8576252

[B23] Crack J Green J Thomson AJ Mechanism of oxygen sensing by the bacterial transcription factor fumarate-nitrate reduction (FNR) J Biol Chem 2004 279 9278 9286 10.1074/jbc.M309878200 14645253

[B24] Khoroshilova N Beinert H Kiley PJ Association of a polynuclear iron-sulfur center with a mutant FNR protein enhances DNA binding Proc Natl Acad Sci U S A 1995 92 2499 2503 10.1073/pnas.92.7.2499 7708673 PMC42245

[B25] Lazazzera BA Bates DM Kiley PJ The activity of the *Escherichia coli* transcription factor FNR is regulated by a change in oligomeric state Genes Dev 1993 7 1993 2005 10.1101/gad.7.10.1993 8406003

[B26] Popescu CV Bates DM Beinert H Munck E Kiley PJ Mössbauer spectroscopy as a tool for the study of activation/inactivation of the transcription regulator FNR in whole cells of *Escherichia coli* Proc Natl Acad Sci U S A 1998 95 13431 13435 10.1073/pnas.95.23.13431 9811817 PMC24836

[B27] Jordan PA Thomson AJ Ralph ET Guest JR Green J FNR is a direct oxygen sensor having a biphasic response curve FEBS Lett 1997 416 349 352 10.1016/S0014-5793(97)01219-2 9373183

[B28] Sutton VR Mettert EL Beinert H Kiley PJ Kinetic analysis of the oxidative conversion of the [4Fe-4S]^2+^ cluster of FNR to a [2Fe-2S]^2+^ Cluster J Bacteriol 2004 186 8018 8025 10.1128/JB.186.23.8018-8025.2004 15547274 PMC529072

[B29] Ullrich S Schüler D Cre-*lox*-based method for generation of large deletions within the genomic magnetosome island of *Magnetospirillum gryphiswaldense* Appl Environ Microbiol 2010 76 2349 2444 10.1128/AEM.02805-09 PMC2849187 20173068

[B30] Kiley PJ Reznikoff WS Fnr mutants that activate gene-expression in the presence of oxygen J Bacteriol 1991 173 16 22 1898918 10.1128/jb.173.1.16-22.1991 PMC207150

[B31] Cruz-Garcia C Murray AE Rodrigues JLM Gralnick JA McCue LA Romine MF Loffler FE Tiedje JM Fnr (EtrA) acts as a fine-tuning regulator of anaerobic metabolism in *Shewanella oneidensis* MR-1 BMC Microbiol 2011 11 64 10.1186/1471-2180-11-64 21450087 PMC3078092

[B32] Bates DM Popescu CV Khoroshilova N Vogt K Beinert H Munck E Kiley PJ Substitution of leucine 28 with histidine in the *Escherichia coli* transcription factor FNR results in increased stability of the [4Fe-4S]^2+^ cluster to oxygen J Biol Chem 2000 275 6234 6240 10.1074/jbc.275.9.6234 10692418

[B33] Zhou G Yin J Chen H Hua Y Sun L Gao H Combined effect of loss of the *caa*_3_ oxidase and Crp regulation drives *Shewanella* to thrive in redox-stratified environments ISME J 2013 7 1752 1763 10.1038/ismej.2013.62 23575370 PMC3749501

[B34] Li YJ Raschdorf O Silva KT Schüler D The terminal oxidase *cbb*_3_ functions in redox control of magnetite biomineralization in *Magnetospirillum gryphiswaldense* J Bacteriolin press 10.1128/JB.01652-14 PMC4097590 24794567

[B35] Bazylinski DA Williams T Schüler D Ecophysiology Of Magnetotactic Bacteria Magnetoreception And Magnetosomes In Bacteria 2006 Heidelberg: SpringerVerlag

[B36] Bates DM Lazazzera BA Kiley PJ Characterization of FNR* mutant proteins indicates two distinct mechanisms for altering oxygen regulation of the *Escherichia coli* transcription factor FNR J Bacteriol 1995 177 3972 3978 7608069 10.1128/jb.177.14.3972-3978.1995 PMC177126

[B37] Sharrocks AD Green J Guest JR *In vivo* and *in vitro* mutants of FNR the anaerobic transcriptional regulator of *E. coli* FEBS Lett 1990 270 119 122 10.1016/0014-5793(90)81248-M 2226775

[B38] Melville SB Gunsalus RP Mutations in *fnr* that alter anaerobic regulation of electron transport-associated genes in *Escherichia coli* J Biol Chem 1990 265 18733 18736 2229038

[B39] Wunsch P Zumft WG Functional domains of NosR, a novel transmembrane iron-sulfur flavoprotein necessary for nitrous oxide respiration J Bacteriol 2005 187 1992 2001 10.1128/JB.187.6.1992-2001.2005 15743947 PMC1064061

[B40] Schüler D Baeuerlein E Dynamics of iron uptake and Fe_3_O_4_ biomineralization during aerobic and microaerobic growth of *Magnetospirillum gryphiswaldense* J Bacteriol 1998 180 159 162 9422606 10.1128/jb.180.1.159-162.1998 PMC106862

[B41] Sambrook J Russel D Molecular Cloning: A Laboratory Manual 2001 3 Cold Spring Habor, New York: Cold Spring Harbor Laboratory Press

[B42] Heermann R Zeppenfeld T Jung K Simple generation of site-directed point mutations in the *Escherichia coli* chromosome using Red^R^/ET^R^ Recombination Microb Cell Fact 2008 7 14 10.1186/1475-2859-7-14 18435843 PMC2373285

[B43] Raschdorf O Müller FD Posfai M Plitzko JM Schüler D The magnetosome proteins MamX, MamZ and MamH are involved in redox control of magnetite biomineralization in *Magnetospirillum gryphiswaldense* Mol Microbiol 2013 89 872 886 10.1111/mmi.12317 23889511

[B44] Viollier E Inglett PW Hunter K Roychoudhury AN Van Cappellen P The ferrozine method revisited: Fe (II)/Fe (III) determination in natural waters Appl Geochem 2000 15 785 790 10.1016/S0883-2927(99)00097-9

