# Supplementary material for: The oxygen sensor MgFnr controls magnetite biomineralization by regulation of denitrification in Magnetospirillum gryphiswaldense
Source: BMC Microbiol. 2014 Jun 10;14:153. doi: 10.1186/1471-2180-14-153 (PMC4065386; doi:10.1186/1471-2180-14-153)
Supplement: Additional file 1 — Magnetosome formation in WT overexpressing MgFnr. Plasmid pLYJ110 and pLYJ153 contains fnr gene from MSR-1 and E. coli, respectively. Cells were grown in anaerobic nitrate medium. Bar, 100 nm. [file 1471-2180-14-153-S1.pdf]

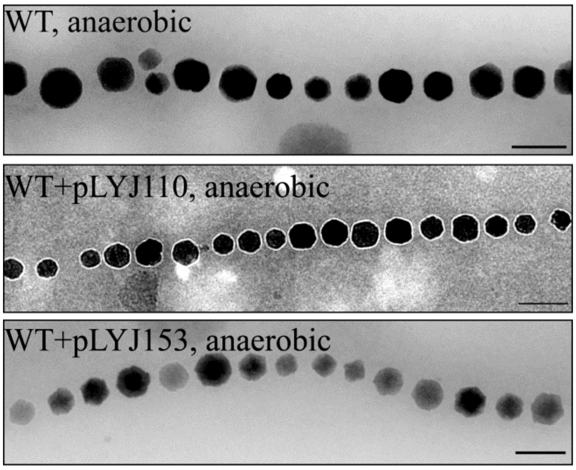

Additional file 1: Magnetosome formation in WT overexpressing MgFnr. Plasmid pLYJ110 and pLYJ153 contains *fnr* gene from MSR-1 and *E. coli*, respectively. Cells were grown in anaerobic nitrate medium. Bar, 100 nm.
