# Supplementary material for: The oxygen sensor MgFnr controls magnetite biomineralization by regulation of denitrification in Magnetospirillum gryphiswaldense
Source: BMC Microbiol. 2014 Jun 10;14:153. doi: 10.1186/1471-2180-14-153 (PMC4065386; doi:10.1186/1471-2180-14-153)
Supplement: Additional file 2 — Detection of Fnr binding sites in the upstream regions of nap , nirS , nor , and nosZ . The putative Fnr binding sites in the promoter regions are indicated in yellow. [file 1471-2180-14-153-S2.pdf]

Additional file 2: Detection of Fnr binding sites in the upstream regions of *nap*, *nirS*, *nor*, and *nosZ*. The putative Fnr binding sites in the promoter regions are indicated in yellow.

**>MGR_4000_5' *nap* operon**<br>ATGTGGACCACAATCCCAAGACCGCCGAGATCGCCCGCGCCATCATCGGCCTGAGTCGCGGCCTC<br>AACCTGGAAGTGGTGGCCGAGGGCTGCGAGATCGCCGCCCATATCCAGTTCCTCAAGGATAATGG<br>CTGCGACACTGTGCAGGGGTTCTTCTATTCCCGACCGGTTCCGGCAGAGCAATTCCAGGCCATGCT<br>GGCCGATGGTTTCCTGCGCGGCCAAGCCTGAGCGGCTAAATCACCAATCACTGATATTTGAATAAC<br>CAAGTAACGGGGTCGGAAAAGACCCTGCTTACTGTGTGGTCTTCTTGACCAAAATCAAATGCGATT<br>CGGACCGATGGGTCCACTCTGGCGGTGCCGGCTCTTCCGTTCGGGAAGGCCTGCGATTACGGGGCC<br>GTTATG
**>MGR_1052_5' *nirS***<br>CCCCGAACAGGGACGCCCCCAGTATTTTCATTTTGGACAGCCGGCTGAACAGTGACTGATTGGAAG<br>CCATGGCTTTAACCCCGAGGGTCTCCTTTTATTCCGGCATTTTATGGCGCTTGTAACACGCCCCCCT<br>AGACCTGAACTTGACGTTGGACAAACCGTCACCGCCAATCACGGCCAGTGCCCAGACCCTATCCC<br>GACCTGCCTGTTGGACTTTGTGACATTGGTCAATTTGGCTTTGACGCATCAAATGCTGATGTTCGTC<br>TGCCGATGACTTTTCGATCGCCTATTTGACTTTGGTTAAAGTCGCTGGCCGACCAAGCGCCCAAGG<br>TGGCCCCACTCGATACGCCGTGCTCTCAAAGGGGGGAGCGGAGCGATCATTATCAAGTGGAGGTA<br>AAGG
**>MGR_3484_5' *nor* operon**<br>GGGCCGATCTTGGCCGCCAGACTGGCCGGCCCCCAGCCGATCTCATCGGCGAACGCGCCCAATCC<br>CAACACTACCACCAGATAGACGATGACCAGCGGCAGCCATCGCATCAGCCCAAAGCCCGCCGGTC<br>CCACCATCAGTGGGATGACGCTGTCGTCCTGACCGGTTCGGCGGTGCCATGGCTTGCGCATGGGCG<br>GCATTTTCGCCGGTGACGCCCGCTTTGTCTCGGTCTTTCGACACCAGCTTGACCAACGTTAAAGCTG<br>ACCAATCGTCTGGGTCATAGGGTCATGGTGACGGGGGAGCCCAAGGTTCCCGCGTGTGAGTTCAG<br>GGTGCGACCACCGGCGGGGGGAGTTTCCGCGGTGGGTTGATCTCAAACCCTTGGAAACCCTTAGG<br>AGCGACC
**>MGR_2761_5' *nosZ***<br>CCCAGCAGATCAAGGGCGGCAGCCATGGTCGACAATGCGGTCGGGCAGACGTCGGGGCAGAAGG<br>TGTAGCCGAAGGCCATCATCCGCACCTTGCCCTTGAAGCTTTCGTCATTGACCCGCTTGCCGTCGTG<br>GGTTTCCAGCAGGAAACGGCCTGAAAACTCCTGGGCTTGTGCGGGCAGTGCCGTCAAAGCCAGGG<br>CGCCAGCCAGCATCAGGGAACCGAGCTTCATCACGTGCACTCCGCTTGCCGTCATCGTTGAAGGCA<br>GTGAAGCAGATCACCGTTTCACGCGACTTGATCAAGGTCACGGCTGGGTCATTAGCGGGCGCGCA<br>TGTTTGCTGGCGAAGTGTTTGGGTAACTCTGTCCCCTTTTAACAGAAACAAGGTGGGGAAAATGAA<br>TCGTACA
