# Supplementary material for: The oxygen sensor MgFnr controls magnetite biomineralization by regulation of denitrification in Magnetospirillum gryphiswaldense
Source: BMC Microbiol. 2014 Jun 10;14:153. doi: 10.1186/1471-2180-14-153 (PMC4065386; doi:10.1186/1471-2180-14-153)
Supplement: Additional file 3 — Transcription of nosZ fused to gusA in Mgfnr variant strains under microaerobic in the presence of nitrate. Expression was measured by β-glucuronidase activity. [file 1471-2180-14-153-S3.pdf]

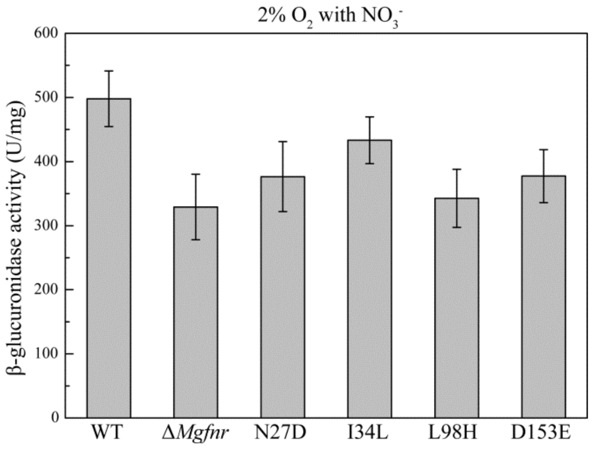

Additional file 3: Transcription of *nosZ* fused to *gusA* in *Mgfnr* variant strains under microaerobic in the presence of nitrate. Expression was measured by β-glucuronidase activity.
