# Supplementary material for: The oxygen sensor MgFnr controls magnetite biomineralization by regulation of denitrification in Magnetospirillum gryphiswaldense
Source: BMC Microbiol. 2014 Jun 10;14:153. doi: 10.1186/1471-2180-14-153 (PMC4065386; doi:10.1186/1471-2180-14-153)
Supplement: Additional file 4 — Magnetosome formation in different Mgfnr variant strains. Cells were grown in microaerobic nitrate medium. Bar, 100 nm. Irregular shaped particles are indicated by black arrows. [file 1471-2180-14-153-S4.pdf]

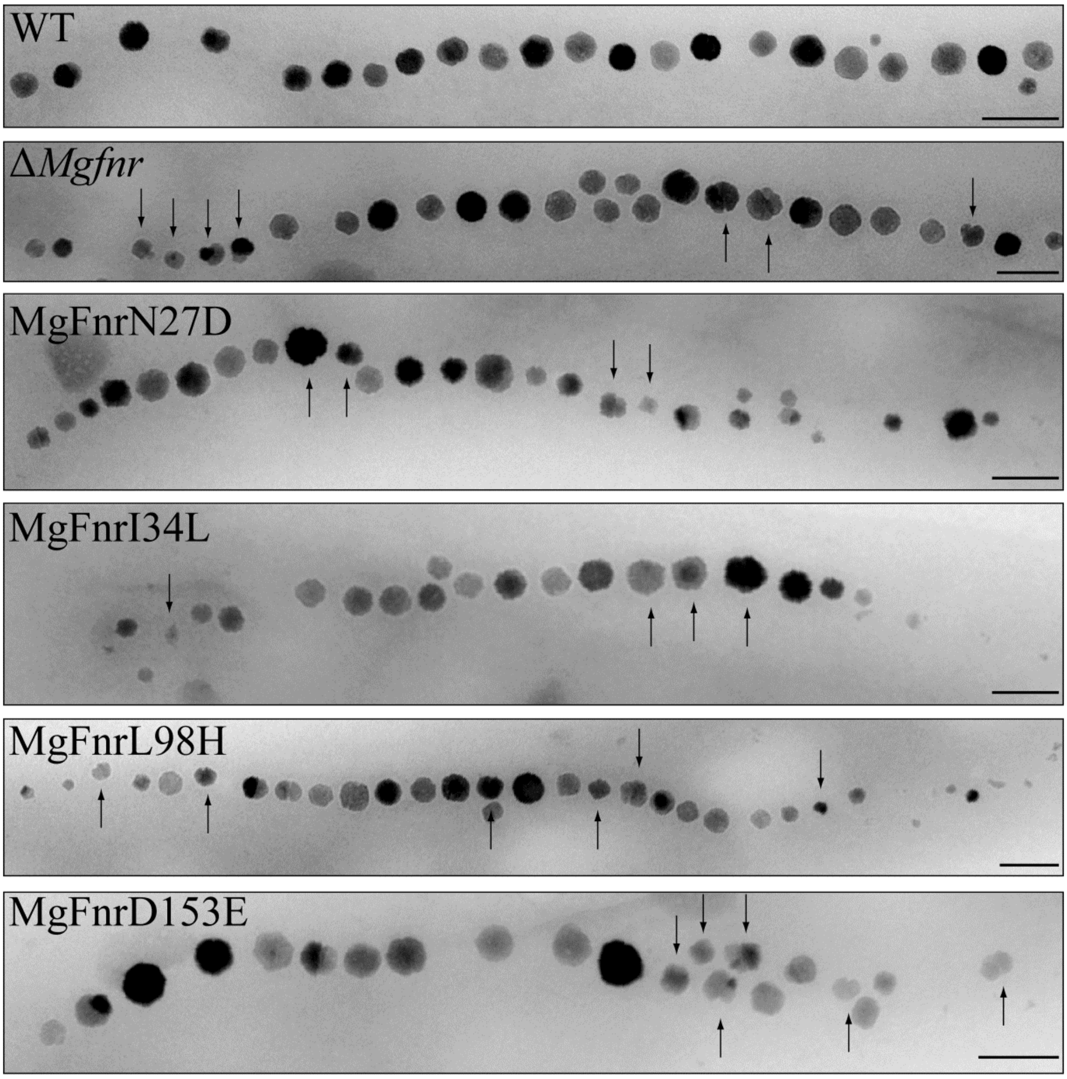

Additional file 4: Magnetosome formation in different *Mgfnr* variant strains. Cells were grown in microaerobic nitrate medium. Bar, 100 nm. Irregular shaped particles are indicated by black arrows.
