# Supplementary material for: The oxygen sensor MgFnr controls magnetite biomineralization by regulation of denitrification in Magnetospirillum gryphiswaldense
Source: BMC Microbiol. 2014 Jun 10;14:153. doi: 10.1186/1471-2180-14-153 (PMC4065386; doi:10.1186/1471-2180-14-153)
Supplement: Additional file 5 — Bacterial strains and plasmids used in this work. [file 1471-2180-14-153-S5.pdf]

Additional file 5: Bacterial strains and plasmids used in this work

Strain or plasmid	Important feature (s)	Source or reference
Strain		
E. coli strain DH5α	F' Φ80d*lacZ*ΔM15Δ(*lacZYA-argF*)*U169 deoR recA1 endA1 hsd*R17 (r_k-, m_k+) *pho*A *sup*E44 λ- *thi*-1 *gyr*A96 *rel*A1	Invitrogen
E. coli strain BW29427	*dap* auxotroph derivative of *E. coli* strain B2155	K. Datsenko and B. L. Wanner, unpublished
Δ*Ecfnr* mutant	*E. coli* K-12 substr. MG1655	K. Jung, unpublished
MSR-1 WT	Wild type R3/S1, but Rif^r, Sm^r	(1)
Δ*Mgfnr*	R3/S1 Δ*Mgfnr*	This study
MgFnrN27D	R3/S1 MgFnrN27D mutants	This study
MgFnrI34L	R3/S1 MgFnrI34L mutant	This study
MgFnrL98H	R3/S1 MgFnrL98H mutant	This study
MgFnrD153E	R3/S1 MgFnrD153E mutant	This study
Plasmid		
pBBR1MCS-2	Km^r, mobilizable broad-host-range vector	(2)
pK19mobGII	-	(3)
pAL01	Km^r, pK19mobGII vector (Km^r, pMB-1 replicon, *gusA*, *lacZ*) containing a 2 kb fragment upstream of *mgr4019*	(4)
pAL02/2	Gm^r, pT18mob2 vector containing a 2 kb fragment downstream of *mgr4019*	(4)
pLYJ87	pBBR1MCS-2 plus *nirS* promoter and *cre* fusion	(5)
pLYJ97	pBBR1MCS-2 plus *gusA* from pK19mobGII	(6)
pOR093	*mamX* CXXCH (65,104)->AXXAH, pK19mobGII derivative, Km^r	(7)
pLYJ105	pAL02/2 plus *Mgfnr* 2-kb upstream region	This study
pLYJ106	pAL01 plus *Mgfnr* 2-kb downstream region	This study
pLYJ109	pLYJ97 plus *Mgfnr* promoter region	This study
pLYJ110	pBBR1MCS-2 plus *Mgfnr* with its own promoter region	This study
pLYJ132	pBBR1MCS-2 plus *Mgfnr*	This study
pLYJ141	pOR093 plus *MgfnrN27D*	This study
pLYJ142	pOR093 plus *MgfnrI34L*	This study
pLYJ143	pOR093 plus *MgfnrD153E*	This study
pLYJ144	pOR093 plus *MgfnrL98H*	This study
pLYJ153	pLYJ36 plus *Ecfnr*	This study

References

1. **Schultheiss D, Kube M, Schüler D.** 2004. Inactivation of the flagellin gene *flaA* in *Magnetospirillum gryphiswaldense* results in nonmagnetotactic mutants lacking flagellar filaments. Appl. Environ. Microbiol. **70:**3624-3631.

2. **Kovach ME, Elzer PH, Hill DS, Robertson GT, Farris MA, Roop RM, Peterson KM.** 1995. Four new derivatives of the broad-host-range cloning vector pBBR1MCS, carrying different antibiotic-resistance cassettes. Gene **166:**175-176.

3. **Katzen F, Becker A, Ielmini MV, Oddo CG, Ielpi L.** 1999. New mobilizable vectors suitable for gene replacement in gram-negative bacteria and their use in mapping of the 3' end of the *Xanthomonas campestris pv.* campestris *gum* operon. Appl. Environ. Microbiol. **65:**278-282.

4. **Lohsse A, Ullrich S, Katzmann E, Borg S, Wanner G, Richter M, Voigt B, Schweder T, Schüler D.** 2011. Functional analysis of the magnetosome island in *Magnetospirillum gryphiswaldense*: the *mamAB* operon is sufficient for magnetite biomineralization. PLoS One **6:**e25561.

5. **Li YJ, Bali S, Borg S, Katzmann E, Ferguson SJ, Schüler D.** 2013. Cytochrome cd_1 nitrite reductase NirS is involved in anaerobic magnetite biomineralization in *Magnetospirillum gryphiswaldense* and requires NirN for proper d_1 heme assembly. J. Bacteriol. **195:**4297-4309.

6. **Li YJ, Katzmann E, Borg S, Schüler D.** 2012. The periplasmic nitrate reductase Nap is required for anaerobic growth and involved in redox control of magnetite biomineralization in *Magnetospirillum gryphiswaldense*. J. Bacteriol. **194:**4847-4856.

7. **Raschdorf O, Müller FD, Posfai M, Plitzko JM, Schüler D.** 2013. The magnetosome proteins MamX, MamZ and MamH are involved in redox control of magnetite biomineralization in *Magnetospirillum gryphiswaldense*. Mol. Microbiol. **89:**872-886.
